# A brief video intervention to promote understanding of the neurobiological base of hazardous alcohol use and treatments

**DOI:** 10.1186/1940-0640-8-S1-A24

**Published:** 2013-09-04

**Authors:** Deborah S Finnell, Shahrzad Nowzari, Mary G Carey

**Affiliations:** 1 Johns Hopkins University School of Nursing, Baltimore, MD, USA; 2 University at Buffalo, School of Nursing, Buffalo, NY, USA; 3 University of Rochester Medical Center, Rochester, NY, USA

## 

The majority (87.4%) of Americans who could benefit from specialty alcohol treatment reported they neither received nor perceived a need for treatment. Common reasons included not being ready to stop consuming alcohol, social stigma, pessimistic attitudes toward the effectiveness of treatment, and attitudinal factors such as minimizing the problem. Disseminating the neuroscience behind alcohol disorders to these at-risk individuals may reduce barriers to acceptance of referral to specialty alcohol treatment. A 20-minute DVD was developed for that purpose. The Transtheoretical Model served as the framework for this brief intervention with active ingredients of the brief intervention corresponding to one of the 10 processes of change. The purpose of this study was to assess the acceptability of, and change in knowledge after the brief intervention. A sample (n=11) of professional firefighters was selected because a previous study among this population found more than half (56%) reported binge drinking (56%) and 14% reported hazardous drinking. A 10-item knowledge test was administered before and immediately after the brief intervention. Mean knowledge scores increased significantly (t=7.72, p < 0.001) from pre- to post-test (43% to 84%, respectively). Acceptability of the video was affirmed from positive comments and interactive discussion following the viewing. This brief alcohol intervention may help shift the current paradigm of shame and blame to one that is consistent with other chronic health disorders (i.e., biologically-based) and otherwise help diminish the stigma that prevents individuals from engaging in alcohol-specialty treatment. This brief intervention is ready to be tested among individuals who are eligible for referral to substance abuse treatment. Intended outcomes will include reduction in alcohol consumption, acceptance of referral to treatment, engagement in treatment, and completion of that specialty treatment.

